# Reduction in membrane component of diffusing capacity is associated with the extent of acute pulmonary embolism

**DOI:** 10.1111/j.1475-097X.2010.01000.x

**Published:** 2011-05

**Authors:** Päivi Piirilä, Mia Laiho, Pirjo Mustonen, Marit Graner, Anneli Piilonen, Merja Raade, Seppo Sarna, Veli-Pekka Harjola, Anssi Sovijärvi

**Affiliations:** 1 Department of Clinical Physiology, HUSLAB, Helsinki University Central HospitalHelsinki, Finland; 2 Department of Acute Care, Malmi Helsinki City HospitalHelsinki, Finland; 3 Department of Haemostasis, Finnish Red Cross Blood ServiceHelsinki, Finland; 4 Department of Medicine, Division of Cardiology, Helsinki University HospitalHelsinki, Finland; 5 Department of Radiology, Helsinki University HospitalHelsinki, Finland; 6 Department of Public Health, University of HelsinkiHelsinki, Finland; 7 Department of Medicine, Division of Emergency Care, Helsinki University HospitalHelsinki, Finland

**Keywords:** diffusing capacity, diffusing capacity of membrane, pulmonary capillary blood volume, pulmonary embolism, right ventricular dysfunction

## Abstract

Acute pulmonary embolism (PE) often decreases pulmonary diffusing capacity for carbon monoxide (DL,CO), but data on the mechanisms involved are inconsistent. We wanted to investigate whether reduction in diffusing capacity of alveolo-capillary membrane (DM) and pulmonary capillary blood volume (Vc) is associated with the extent of PE or the presence and severity of right ventricular dysfunction (RVD) induced by PE and how the possible changes are corrected after 7-month follow-up. Forty-seven patients with acute non-massive PE in spiral computed tomography (CT) were included. The extent of PE was assessed by scoring mass of embolism. DL,CO, Vc, DM and alveolar volume (VA) were measured by using a single breath method with carbon monoxide and oxygen both at the acute phase and 7 months later. RVD was evaluated with transthoracic echocardiography and electrocardiogram. Fifteen healthy subjects were included as controls. DL,CO, DL, CO/VA, DM, vital capacity (VC) and VA were significantly lower in the patients with acute PE than in healthy controls (*P*<0·001). DM/Vc relation was significantly lower in patients with RVD than in healthy controls (*P* = 0·004). DM correlated inversely with central mass of embolism (*r* = −0·312; *P* = 0·047) whereas Vc did not. DM, DL,CO, VC and VA improved significantly within 7 months. In all patients (*P* = 0·001, *P* = 0·001) and persistent RVD (*P* = 0·020, *P* = 0·012), DM and DL,CO remained significantly lower than in healthy controls in the follow-up. DM was inversely related to central mass of embolism. Reduction in DM mainly explains the sustained decrease in DL,CO in PE after 7 months despite modern treatment of PE.

## Introduction

Acute pulmonary embolism (PE) is known to be associated with decreased pulmonary diffusing capacity for carbon monoxide (DL,CO) (Sharma *et al.*, 1980) reflecting deterioration of gas exchange capacity of the lungs. However, the mechanisms behind decreased DL,CO in PE are still in many ways obscure.

The transfer of carbon monoxide (CO) through the alveolo-capillary membrane depends on the diffusing capacity of the membrane itself (DM), the pulmonary capillary blood volume (Vc) and haemoglobin concentration of blood. Some earlier studies have suggested that the reduction in DL,CO in PE would be owing to loss of alveolar volume (VA) (Pande *et al.*, 1975; Fennerty *et al.*, 1988; Wimalaratna *et al.*, 1989) rather than owing to decrease in Vc (Sharma *et al.*, 1980). Other authors have proposed that reduction in DM related to Vc in chronic thromboembolic pulmonary hypertension (Bernstein *et al.*, 1996) or in chronic thromboembolic disease (Oppenheimer *et al.*, 2006) would cause the reduced DL,CO. Some have found in chronic thromboembolic hypertension both Vc and DM to be reduced (Steenhuis *et al.*, 2000). Thus, the previous studies have resulted in conflicting results.

PE displays a wide spectrum of clinical severity, depending on the size and distribution of the embolic mass. In high-risk and intermediate-risk cases of PE (massive and submassive PE), right ventricular dysfunction (RVD) is observed.

The aim of the study was to analyse changes in the components of diffusing capacity in acute PE. We measured DL,CO, DM, Vc and lung volume and correlated them with the indices of the extent of PE. The measurements were repeated after 7 months. Our aim was also to assess whether reduction in diffusing capacity and its components were associated with the extent of PE (measured as either total, central or peripheral mass of embolism), or with PE-induced RVD. For comparison, healthy control subjects were also studied. In addition, the patients were re-evaluated after 7 months to see how the possible changes would be corrected after evidence-based therapy.

## Methods and materials

### Patients and study design

We studied 47 consecutive patients (24 women, 23 men) with acute non-massive, non-high-risk PE confirmed by computerized tomography (CT) at the Emergency Department of Helsinki University Central Hospital. Patients were recruited between January 2003 and August 2004. Exclusion criteria comprised clinically massive PE (haemodynamically unstable patients), chronic pulmonary disease requiring regular medication, previous PE, non-stable angina pectoris, patients on anticoagulation therapy and terminal cancer (with estimated life expectancy of less than 7 months). In addition, patients arriving at the hospital between Friday 18:00 and Sunday 12:00 were excluded because the services of the laboratory of clinical physiology were unavailable at that time. For those included in the study, echocardiography (ECHO) and lung function studies were performed.

Patients were given antithrombotic treatment according to current guidelines at the time of the study (Torbicki *et al.*, 2000). Most of the patients were treated initially with low molecular weight heparin, followed by warfarin for ≥6 months. Seven months later, diffusing capacity measurements were repeated and flow-volume spirometry was performed. ECHO was also repeated to identify patients with RVD.

Fifteen healthy, age-matched controls (nine women, six men) were also studied – in 12 of them (three of the controls did not cooperate to come to the second measurement), the measurements were repeated later, correspondingly to the patients. The anthropometric data and smoking history of the patients and healthy controls are presented in Table 1.

**Table 1:** The gender and anthropometric data of the patients with pulmonary embolism (PE) and the healthy control subjects

	Patients with PE	Healthy controls	Significance in *t*-test
Gender, male/female (number)	23/24	6/9	
Weight (kg)	88·9 (18·1)	73·0 (11·2)	0·002
Height (cm)	172·0 (10·4)	173·0 (11·1)	0·730
Age (year)	56·1 (16·3)	55·0 (16·5)	0·881
Smoking (pack-years)	3·92 (7·6)	2·67 (6·0)	0·469
Number (percent) of			
non-smokers	30 (63·8)	11 (73·3)	0·761*
smokers	7 (14·9)	2 (13·3)	
exsmokers	10 (21·3)	2 (13·3)	

* Chi-square test.

The study protocol was approved by the ethics committee of Helsinki University Hospital, and informed consent was signed by all participants.

### Lung function methods

Measurements of DL,CO with DM and Vc determinations were performed within 24 h from PE diagnosis. For measurement of DL,CO, single breath method according to Viljanen (1982) was used applying the European Respiratory Society recommendations (McIntyre *et al.*, 2005). Slow vital capacity (VC) was measured first, and the inspiratory volume for 10-s breath holding was standardized to 90% of measured VC. At least two successive determinations were performed, and the mean value was recorded for analysis. In addition, VC, VA and specific diffusing capacity (DL,CO/VA) were determined (McIntyre *et al.*, 2005). DM, Vc and rate of uptake of CO by red cells per mmHg of CO tension (θ) were determined according to Roughton and Forster (1957; Roughton *et al.*, 1957;). Two different gas mixtures were used (i) oxygen 21%, helium 7%, CO 0·25% and nitrogen the rest and (ii) oxygen 93%, helium about 7% and CO 0·25%. The measurements were accomplished with a Jaeger MasterScreen PFT equipment (Würzburg, Germany). Diffusing capacity values were corrected according to actual haemoglobin concentration in blood (McIntyre *et al.*, 2005). Reference values of Viljanen (1982) for Finnish population were used for DL,CO and DL,CO/VA values and those of Zanen *et al.* (2001) for DM and Vc values.

To exclude patients with pulmonary obstruction, flow-volume spirometry was performed on PE patients 7 months after the acute phase in the patients and at the first visit from the control subjects with a pneumotachograph connected with a microcomputer (Medikro MR-3; Medikro, Kuopio, Finland) (Table 2). For safety reasons, forced spirometry was not performed at the acute phase. Reference values of Viljanen (1982) were used. The flow-volume curve for analysis was compiled from at least three successive forced expiratory curves using the envelope method according to the European Respiratory Society (Quanjer Ph *et al.*, 1993) guidelines. The following variables were measured: forced vital capacity (FVC), forced expiratory volume in one second (FEV1) and forced expiratory flow at a level where 50% of FVC remains to be exhaled (MEF50).

**Table 2:** Spirometric data of patients and healthy controls. ANCOVA, used adjustment thoroughly in the methods chapter

	Patients (*N* = 47)	Controls (*N* = 15)	
			
Variable	Mean (SD)	Mean (SD)	Significance of the difference
FVC (l)	3·6 (1·2)	4·1 (1·1)	0·167
FVC (%)	88·3 (14·4)	101·2 (17·1)	0·039
FEV1 (l)	2·9 (0·93)	3·4 (0·88)	0·051
FEV1 (%)	88·0 (15·0)	97·9 (28·3)	0·098
FEV1/FVC (%)	80·8 (5·3)	83·1 (6·3)	0·180
MEF 50 (l s^−1^)	3·2 (1·4)	4·2 (1·3)	0·050
MEF50 (%)	74·8 (26·7)	95·7 (27·9)	0·014

FVC, forced vital capacity; VC, vital capacity.

### Quantifying the extent of pulmonary embolism and right ventricular dysfunction

Spiral computerized tomography (CT) angiographic studies of pulmonary arteries were performed with an 8-slice, 4-slice or single-slice scanner in 27, 18 and 2 patients, respectively. The volume of contrast material varied between 90 and 120 ml and was injected with a power injector using bolus tracking. Slice thickness was 1 or 1·25 mm in the multi-slice scanners and 3 mm in the single-slice scanner. Studies were analysed on a CT workstation by two experienced radiologists, using different window settings and 2D reformations when needed. Mass of embolism was scored using the method of Mastora *et al.* (2003), in which the percentage of obstructed surface of each artery is evaluated using a 5-point scale. The score for mass of embolism is 0–55 for central emboli (thrombus in mediastinal or lobar arteries) and 0–100 for peripheral (segmental) arteries. The total score for mass of embolism is thus 0–155. If the embolism was only in subsegmental arteries, the count was 0.

Transthoracic ECHO was performed within 24 h of PE diagnosis. The presence of RVD was assessed using established echocardiographic criteria: (i) increased right ventricular to left ventricular (RV/LV) ratio of end-diastolic diameter (>0·9) at the left parasternal long axis, (ii) wall-motion abnormality of the interventricular septum and (iii) peak velocity of tricuspid regurgitation >2·8 m/s (Ribeiro *et al.*, 1997; Torbicki *et al.*, 2000; Goldhaber, 2002). At least one of these three criteria had to be positive. ECHO was performed by one of three experienced cardiologists blinded to the results of biochemical assays. A 12-lead ECG at rest was also recorded. Criteria for signs of right ventricular overload in ECG were T-wave inversion in leads V1-V3, incomplete or complete right bundle branch block, S1Q3T3 or signs of right atrial enlargement (Geibel *et al.*, 2005, Punukollu *et al.*, 2005).

### Statistical methods

The lung function variables were compared between patients and controls with independent samples t-test. Correlation methods were used to determine the covariants, and the results were adjusted for them by using general linear model procedure analysis of covariance (ANCOVA). For the variables used as per cent of predicted values, the results were adjusted to pack years and also to weight if weight was not calculated in the reference value; for the variables used as absolute values, the results were adjusted height, weight, age and smoking. In multiple comparisons, Bonferroni correction was used. In text, the results are reported as absolute values, because for all variables, reference values were not available. In addition, results are reported as per cent of reference values in the tables.

Partial correlation was used to adjust the lung function variables for age, height, weight and smoking. If the variables were dealt with as per cent of reference values, they were adjusted for smoking. Also weight and height were adjusted for if they were not included in the reference values.

The change in the studied variables during follow-up was analysed with paired t-test or with Wilcoxon's pairwise test when the variable was not normally distributed. To evaluate the influence of lung volume on the observed changes, the difference in VA between the acute and recovery phases of PE was compared in regression analysis with the corresponding difference in DL,CO, DL,CO/VA, DM or Vc between the examination phases. spss Windows version 15.0 (IBM Corporation, Somers, NY, USA) was used in all calculations.

## Results

### Acute phase

The total score for mass of embolism varied between 0 and 110 (mean, SD; 54·1, 32·4), corresponding 0–71% obstruction of the total pulmonary artery bed. Forty patients had both central and peripheral emboli. Seven patients had peripheral emboli only and one of them subsegmental emboli only, resulting in score 0 in that case. The score for central mass of embolism varied 0–34 (mean, SD; 17·5, 1·8), corresponding 0–62% obstruction of central pulmonary artery bed. The scores for peripheral mass of embolism ranged from 0 to 82 (37·0, 22·5), meaning 0–82% obstruction of peripheral pulmonary artery bed. Signs of RVD in ECHO were present in 24 patients (51%), in CT 29 (63%) or in ECG 23 (42·6%). The comparisons between patients with and without RVD are given based on ECHO examination, because ECHO examination was available also at the control phase. Associated with PE, two patients (3·7%) had findings of pulmonary infarction, four patients (7·4%) atelectasis and four patients (7·4%) pleural fluid; all these findings were slight.

VC, VA, DL,CO, DL,CO/VA and DM were significantly lower in patients with acute PE than in the healthy controls (Table 3). In patients, DL,CO (*r* = 0·708; *P*<0·001), DM (0·447, *P* = 0·003) and Vc (*r* = 0·502, *P* = 0·001) correlated significantly with VA.

**Table 3:** Lung function data of patients and the healthy controls during the acute phase and at the recovery phase (7-month later). Mean and standard deviations are presented for all variables

Variable	Patients, acute Phase *N* = 47	Controls, acute phase, *N* = 15	Statistical significance of comparison between patients and controls, acute phase (*P*-value)^a^,^b^	Patients, recovery phase *N* = 47	Statistical significance of comparison of patients between acute and recovery phases (*P*-value)^a^,^c^	Controls, recovery phase *N* = 12	Statistical significance of comparison between patients and controls, recovery phase (*P*-value)^a^,^b^
VC (l)	3·5 (1·1)	4·4 (1·1)	0·022	3·80 (1·19)	<0·001	4·25 (1·17)	0·514
VC (%)	83·3 (15·5)	103·9 (14)	<0·001	90·3 (16·4)	<0·001	104·1 (15·2)	0·478
VA (l)	4·3 (1·1)	5·5 (1·3)	0·002	4·7 (1·2)	<0·001	5·1 (1·15)	0·432
DL,CO (mmol min^−1^kPa^−1^)	5·9 (1·8)	8·3 (2·5)	<0·001	6·6 (2·1)	<0·001	8·2 (2·5)	0·001
DL,CO (%)	73·7 (13·6)	103·7 (15·2)	<0·001	81·3 (14·4)	<0·001	101·1 (15·59)	0·003
DL,CO/VA (mmol min^−1^kPa^−1^ l^−1^)	1·39 (0·2)	1·48 (0·2)	0·008	1·30 (0·24)	0·568	1·47 (0·27)	0·012
DL,CO/VA (%)	94·6 (14·5)	104·4 (13·7)	0·032	96·3 (14·4)	0·278	104·8 (17·2)	0·025
DM (mmol min^−1^ kPa^−1^)	9·1 (3·0)	13·9 (4·7)	<0·001	10·3 (3·8)	0·001	13·6 (4·9)	0·001
DM (%)	49·3 (15·6)	72·3 (22·8)	<0·001	55·0 (19·3)	0·003	71·7 (28·7)	0·008
Vc (ml)	53·6 (16·1)	63·9 (22·6)	0·157	56·6 (16·5)	0·159	69·3 (19·5)	0·040
Vc (%)	68·7 (17·2)	79·3 (19·4)	0·062	72·0 (16·5)	0·173	85·8 (18·1)	0·049
DM/Vc relation	0·18 (0·09)	0·22 (0·06)	0·131	0·19 (0·09)	0·770	0·21 (0·11)	0·344

VA, alveolar volume; VC, vital capacity; DM, diffusing capacity of alveolo-capillary membrane; Vc, pulmonary capillary blood volume.

a Level of significance according to Bonferroni correction is 0·004.

b non-paired ANCOVA, adjustments are thoroughly given in the methods chapter.

c paired *t*-test.

**Figure 1:**
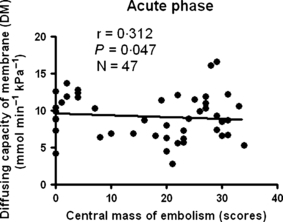
Association of the diffusing capacity of the alveolo-capillary membrane with central embolism mass. The calculation was made with partial correlation with adjustment with age, smoking at pack years, height and weight.

DM did not correlate significantly with total or peripheral mass of embolism, but scores for central mass of embolism showed a significant negative correlation with DM (Fig. 1). Vc did not correlate significantly with scores for mass of embolism.

DL,CO/VA was suggestively lower in those with RVD than in those without RVD. VC, VA, DL,CO, DL,CO/VA, DM and DM/Vc relations were significantly lower in patients with RVD compared with healthy controls. VC, VA, DL,CO and DM were significantly lower in patients without RVD compared with healthy controls (Table 4).

**Table 4:** The results of patients with and without right ventricular dysfunction (RVD) in echocardiography in the acute phase of pulmonary embolism. Also the comparisons of them with healthy controls are presented (non-paired ANCOVA)

Variable	Patients without RVD (*N* = 23)	Patients with RVD (*N* = 24)	Healthy controls (*N* = 15)	Statistical significance of comparison between patients with and without RVD (*P*-value)^a^,^b^	Statistical significance of comparison between patients with RVD and controls (*P*-value)^a^,^b^	Statistical significance of comparison between patients without RVD and controls (*P*-value)^a^,^b^
VC (l)	3·5 (1·2)	3·4 (1·0)	4·4 (1·1)	0·618	0·013	0·064
VC (%)	83·3 (18·4)	83·2 (13·5)	103·9 (14)	0·988	<0·001	0·001
VA (l)	4·37(1·17)	4·27 (0·98)	5·5 (1·3)	0·596	0·006	0·012
DL,CO (mmol min^−1^ kPa^−1^)	6·3 (1·9)	5·67 (1·7)	8·3 (2·5)	0·218	<0·001	0·002
DL,CO (%)	76·8 (14·2)	71·0 (13·3)	103·7 (15·2)	0·139	<0·001	<0·001
DL,CO/VA (mmol min^−1^kPa^−1^ l^−1^)	1·46 (0·25)	1·32 (0·23)	1·48 (0·2)	0·015	0·009	0·062
DL,CO/VA (%)	94·6 (14·5)	104·4 (13·7)	104·4 (13·7)	0·025	0·007	0·293
DM (mmol min^−1^ kPa^−1^)	9·7 (3·0)	8·5 (3·0)	13·9 (4·7)	0·185	<0·001	0·001
DM (%)	51·7 (16·4)	46·4 (14·5)	72·3 (22·8)	0·383	<0·001	0·003
Vc (ml)	55·8 (17·6)	51·4 (14·6)	63·9 (22·6)	0·446	0·333	0·211
Vc (%)	70·6 (18·9)	66·0 (15·9)	79·3 (19·4)	0·441	0·043	0·195
DM/Vc relation	0·19 (0·12)	0·17 (0·06)	0·22 (0·06)	0·484	0·004	0·674

VA, alveolar volume; VC, vital capacity; DM, diffusing capacity of alveolo-capillary membrane; Vc, pulmonary capillary blood volume.

a Level of significance according to Bonferroni correction is 0·004.

b non-paired ANCOVA, adjustments are thoroughly explained in the methods chapter.

### Recovery phase (7 months)

In patients, most lung function variables improved during 7-month follow-up (Table 3), the increase in DM was in mean 14·3% (SD 28%), *P*<0·001, and in Vc 9·96% (SD 35%) *P* = 0·159 (Table 3). The increase in VA explained the improvement in DL,CO (*P* = 0·008) and DM (*P* = 0·029).

DL,CO, DL,CO/VA, DM and Vc remained significantly lower in the patients when compared to the controls (Table 3). In all patients, DL,CO (*r* = 0·782, *P*<0·001), DM (*r* = 0·642; *P*< 0·001) and Vc (*r* = 0·467; *P* = 0·002) correlated significantly with VA.

**Figure 2:**
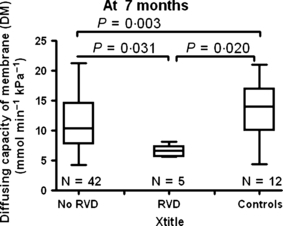
Diffusing capacity of membrane in the recovery phase. A suggestive difference existed between patients with and without right ventricular dysfunction (RVD), a significant difference between patients without RVD and healthy controls, as well as between patients with RVD and healthy controls (ANCOVA, adjustment according to age, height, weight and smoking). The level of significance after Bonferroni correction is 0·02.

**Figure 3:**
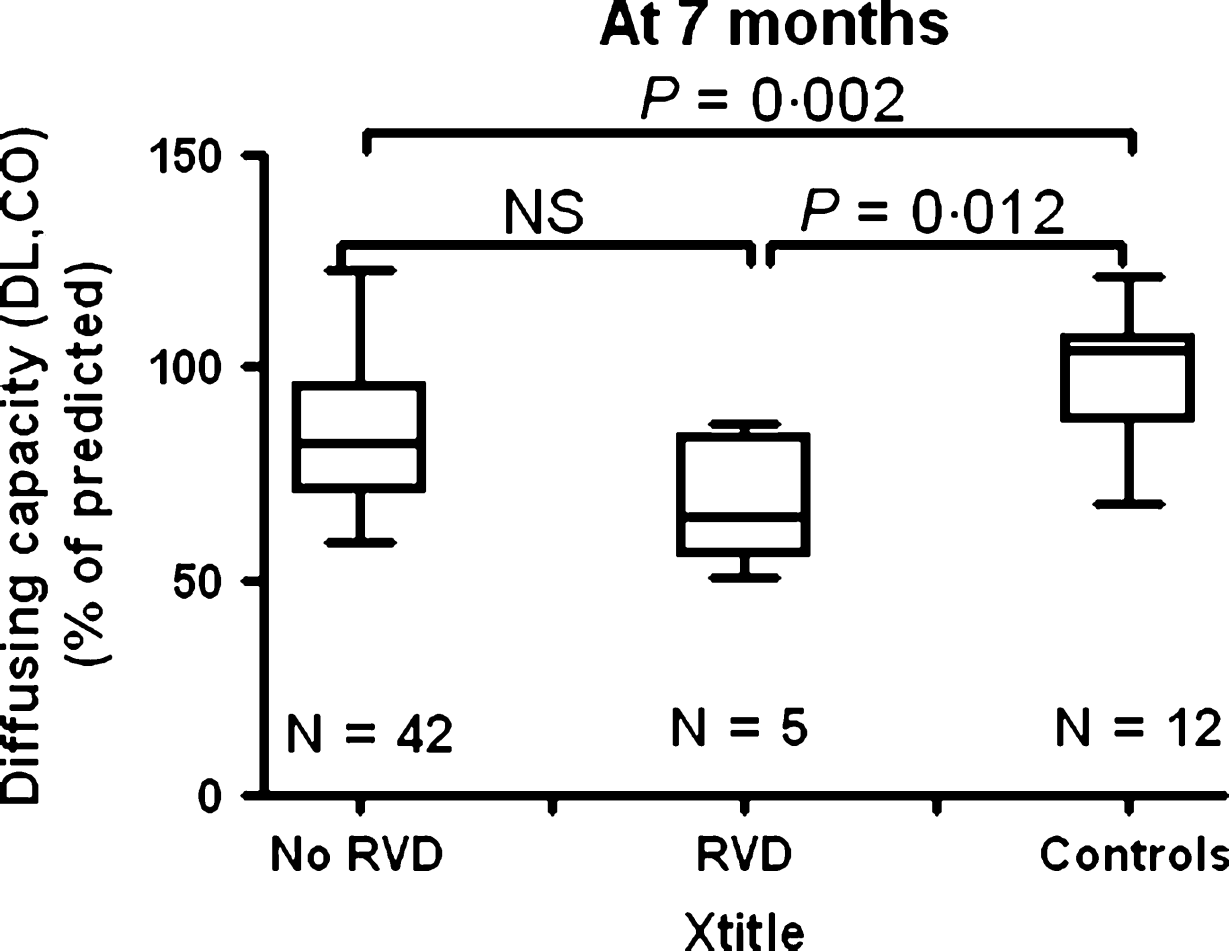
Diffusing capacity (DL,CO) in the recovery phase. A significant difference existed between patients without right ventricular dysfunction (RVD) and healthy controls and between patients with RVD and healthy controls (ANCOVA, adjustment according to smoking). The level of significance after Bonferroni correction is 0·02.

At 7 months, the size and function of the right ventricle had normalized in most patients with RVD at the baseline (24/47, 51%), but still 5/47 patients (10·6%) had RVD in ECHO. In patients with persistent RVD at 7 months, DM was suggestively lower than in those without RVD or significantly lower than in healthy controls (Fig. 2). Also DL,CO remained significantly lower in those with RVD than in healthy controls (Fig. 3). In patients with permanent RVD, the correlations between VA and DL,CO, DL,CO/VA, DM or Vc were not significant.

## Discussion

This study shows that diffusing capacity (DL,CO) and especially its alveolo-capillary membrane component (DM) are decreased in acute PE. The extent of PE and the severity of RVD were inversely associated with DL,CO and DM; the larger the PE, the lower the DL,CO or DM, findings not reported previously. Although DL,CO and DM increased during the 7-month recovery phase, they still remained lower than in healthy control subjects, especially in patients with persistent RVD. In persistent RVD, the reduction in DM seemed to be independent of lung volume.

As far as we know, the present study is the largest follow-up study in PE in which the components of diffusing capacity have been evaluated. Earlier, Fennerty *et al.*(1988) conducted a smaller study including 14 patients with PE with a 3-month follow-up and found DL,CO to be the best in differentiating between patients and controls, and DM to be lower in patients than in controls, but the difference in the latter – in contrary to the present results – did not reach statistical significance. In the present study, more exact characterization of the embolism mass could be obtained than in the previous study (Fennerty *et al.*,1988), which used a ventilation-perfusion isotope scan with only a crude estimation of the size of PE. However, also with CT methods, high correlations were not found, which may depend on the heterogeneity of the embolism mass. As far as we know, the present paper is the first study to use the CT-based scoring of mass of embolism in relation to the components of diffusing capacity.

The results indicate that also the area of the alveolo-capillary membrane affected by thrombus, i.e. the extent of PE, is responsible for reduction in DL,CO at the acute phase of PE, in addition to the effect of reduction in lung volume. Loss of lung volume is important in the mechanism of decreased DL,CO explained by bronchoconstriction in terminal respiratory units corresponding to the site of PE (Nadel *et al.*, 1964).

VC and VA improved significantly 7 months after the acute PE. In the recovery phase, the correlation between DM and VA remained significant in all patients, but in patients with sustained RVD, VA seems to be a less important cause of reduction in DM and DL,CO. In patients with RVD, eventually a decrease in the area of functional alveolar membrane occurs, because of organized thrombus, medial hypertrophy or intimal fibrosis, as described earlier in chronic thromboembolic pulmonary hypertension, a disease more advanced than the disease of the present patients with RVD (Moser & Bloor, 1993). At 7 months, DM probably reflects more the properties of alveolo-capillary membrane whereas in the acute phase, DM reduction might reflect more the mass of embolism. However, the method used in the present study cannot differentiate between the causes of DM reduction. In addition, the small number of those with persistent RVD reduces the power of these findings in the recovery phase.

As a rule, PE causes RVD when at least 25% (Goldhaber, 1998) of the pulmonary arterial bed is occluded by a thrombus. Increase in pulmonary arterial pressure in PE increases right ventricular afterload and consequently leads to dilatation and failure of the right ventricle. The larger the mass of embolism, the more common and severe the RVD. In general, approximately half of the patients with acute PE have RVD (Goldhaber, 1998; Miller *et al.*,1998, Lankeit & Konstantinides, 2010). The prevalence of RVD of 51% in the acute phase of the present study is in line with that.

As it concerns Vc, we did not find a significant difference in its values between healthy controls and patients at the acute phase, which is in concordance to one older study (Fennerty *et al.*,1988). Either did Vc not improve during the follow-up. There are also earlier studies on PE (Fennerty *et al.*, 1988) and chronic thromboembolic disease (Bernstein *et al.*, 1996) which report that Vc did not recover after therapeutic procedures, although there are also suggestions on the role of Vc in the reduction in DL,CO in chronic thromboembolic disease (Steenhuis *et al.*, 2000).

In the present study, DM/Vc was significantly lower in patients with RVD compared with those without RVD, suggesting a role of DM/Vc relation in the pathophysiology of RVD. Reduction in the effective capillary volume in PE has been suggested to cause vascular dilatation, leading to reduction in the DM/Vc ratio (Pande *et al.*, 1975; Oppenheimer *et al.*, 2006). In the present study, there was improvement in both the values of DM and Vc why their relation possibly did not significantly change during the follow-up.

The method used to measure the components of diffusing capacity is a well-established one (Roughton & Forster, 1957), although it has less commonly been utilized in recent years. The reference values of Zanen *et al.* (2001) were chosen because they are the most recent ones.

The levels of Vc and DM in the present study were similar to those in an earlier paper (Fennerty *et al.*, 1988) after the relevant units had been converted. However, the present study gives new information on the mechanism and outcome of PE.

In our hospital, PE is routinely diagnosed by CT, as recommended by international guidelines (Torbicki *et al.*, 2008). Thus, we wanted to study PE according to modern praxis. It would be ideal to study both CT and perfusion scintigraphy. However, this was not possible to arrange and would have been a heavy protocol with a high radiation dose for the patients. In addition, the patients arrived acutely at the hospital, why it was not possible to arrange examination time for both CT and scintigraphy. CT is not as good as scintigraphy on discriminating embolism in small arteries or to analyse peripheral distribution of pulmonary flow. However, in analysing the extent of central, segmental and larger subsegmental emboli, CT is superior to scintigraphy (Remy-Jardin *et al.*, 1996; Mayo *et al.*, 1997).

In our study population, patients with obstructive pulmonary disease were excluded based on spirometry. The FEV1/FVC ratio was normal in both patients and controls, indicating that patients did not have obstruction in medium-sized or large bronchi. Smoking likely had not induced any significant impairment during the 7-month follow-up. In addition, statistical calculations were adjusted for smoking. Some smokers were also present among control subjects. Because there was a significant difference in weight between patients and control subjects, the statistical calculations were adjusted for weight.

In conclusion, a decrease in diffusing properties of the alveolo-capillary membrane for carbon monoxide is associated with the extent of central PE and explains the found sustained reduction in DL,CO in PE. Vc may also be involved in the sustained reduction in DL,CO in PE. The diffusing capacity recovered during the 7-month follow-up but did not reach the level of healthy controls nor the normal reference range. Our data provide new knowledge of consequences of PE on lung function and indicate that sustained reduction in diffusing capacity may still remain despite the treatment of PE according to current guidelines.
